# Enhanced fibrotic potential of COL1A1^hi^NR4A1^low^ fibroblasts in ischemic heart revealed by transcriptional dynamics heterogeneity analysis at both bulk and single-cell levels

**DOI:** 10.3389/fcvm.2024.1460813

**Published:** 2025-01-06

**Authors:** Cheng Luo, Baoping Tan, Luoxiang Chu, Liqiang Chen, Xinglong Zhong, Yangyang Jiang, Yuluan Yan, Fanrui Mo, Hong Wang, Fan Yang

**Affiliations:** ^1^ Department of Cardiology, Liuzhou Workers’ Hospital, The Fourth Affiliated Hospital of Guangxi Medical University, Liuzhou, China; ^2^ Medical Science Research Center, The Fourth Affiliated Hospital of Guangxi Medical University, Liuzhou, China; ^3^ Liuzhou Key Laboratory of Primary Cardiomyopathy in Prevention and Treatment, The Fourth Affiliated Hospital of Guangxi Medical University, Liuzhou, China; ^4^ Department of Oncology, Liuzhou Workers’ Hospital,The Fourth Affiliated Hospital of Guangxi Medical University, Liuazhou, China; ^5^ Rehabilitation Department, Liuzhou Workers’ Hospital,The Fourth Affiliated Hospital of Guangxi Medical University, Liuzhou, China

**Keywords:** fibroblasts, ischemic heart disease, cardiac fibrosis, single nuclei RNA sequencing, diagnostic model

## Abstract

**Background:**

Fibroblasts in the fibrotic heart exhibit a heterogeneous biological behavior. The specific subsets of fibroblasts that contribute to progressive cardiac fibrosis remain unrevealed. Our aim is to identify the heart fibroblast (FB) subsets that most significantly promote fibrosis and the related critical genes as biomarkers for ischemic heart disease.

**Methods:**

The single nuclei RNA sequencing (snRNA-seq) and bulk RNA sequencing datasets used in this study were obtained from the Gene Expression Omnibus (GEO). The activity of gene sets related to progressive fibrosis was quantified for each FB cluster using the AddmoleculeScore function. Differentially expressed genes (DEGs) for the specific cell cluster with the highest fibrotic transcription dynamics were identified and integrated with bulk RNA sequencing data for analysis. Multiple machine learning models were employed to identify the optimal gene panel for diagnosing ischemic heart disease (IHD) based on the intersected DEGs. The effectiveness and robustness of the gene-derived diagnostic tool were validated using two independent IHD cohorts.Subsequently, we validated the signature genes using a rat post-myocardial infarction heart failure model.

**Results:**

We conducted an analysis on high-quality snRNA-seq data obtained from 3 IHD and 4 cardiac sarcoidosis heart samples, resulting in the identification of 16 FB clusters. Cluster2 exhibited the highest gene activity in terms of fibrosis-related transcriptome dynamics. The characteristic gene expression profile of this FB subset indicated a specific upregulation of COL1A1 and several pro-fibrotic factors, including CCDC102B, GUCY1A3, TEX41, NREP, TCAP, and WISP, while showing a downregulation of NR4A1, an endogenous inhibitor of the TGF-*β* pathway. Consequently, we designated this subgroup as COL1A1^hi^NR4A1^low^ FB. Gene set enrichment analysis (GSEA) shows that the gene expression pattern of COL1A1^hi^NR4A1^low^ FB was closer to pathways associated with cardiac fibrosis. Through machine learning, ten feature genes from COL1A1^hi^NR4A1^low^ FB were selected to construct a diagnostic tool for IHD. The robustness of this new tool was validated using an independent cohort and heart failure rats.

**Conclusion:**

COL1A1^hi^NR4A1^low^ FB possess heightened capability in promoting cardiac fibrosis. Additionally, it offers molecular insights into the mechanisms underlying the regulation of the TGF-*β* pathway. Furthermore, the characteristic genes of COL1A1hiNR4A1 FB could serve as valuable tools for diagnosing of IHD.

## Introduction

Heart failure (HF) represents a prevalent clinical syndrome marked by inherent structural and/or functional anomalies in the cardiac system, leading to an excess of 9 million fatalities each year and presenting a prominent global health predicament (1). Coronary artery atherosclerosis-induced ischemic heart disease (IHD) stands as a predominant etiology for HF (2). IHD precipitates a decline in left ventricular function, and contributes to heart failure, regardless of the presence or absence of acute myocardial infarction (3).

Fibrotic extracellular matrix replacement and an imbalance between type I and III collagen fibers—hallmarks of cardiac remodeling—are central to HF pathophysiology in IHD (4, 5). This dysregulation further exacerbates the mechanical repercussions associated with cardiac contraction and relaxation (6). Early left ventricular remodeling, detectable through imaging following myocardial infarction, is closely linked to adverse clinical outcomes, with its severity influenced by the extent of transmural necrosis and the formation and localization of scar tissue (7–9).

Efforts aimed at preventing adverse remodeling and promoting reverse remodeling in patients after myocardial infarction include revascularization, neuroendocrine pathway inhibition, and the implantation of ventricular assist devices (10), but not all patients can benefit from them. Moreover, existing risk assessment methods are insufficient for identify those at high risk of rapid adverse remodeling (11). Exploring new biomarkers and understanding their role in IHD-related heart failure can provide insights into the underlying biological mechanisms.

Collagen, the primary component of the extracellular matrix (ECM), is synthesized by fibroblasts (12). Under normal physiological conditions, fibroblasts remain in a quiescent state, maintaining tissue homeostasis with minimal ECM production. However, During cardiac injury, such as ischemia or mechanical stress, quiescent fibroblasts transform into myofibroblasts, which drive fibrosis by producing excessive ECM and expressing *α*-smooth muscle actin (*α*-SMA). This transformation is primarily regulated by the TGF-*β* pathway, which activates fibroblasts and promotes collagen synthesis (12). Activated myofibroblasts are key contributors to excessive ECM deposition, leading to tissue stiffness and pathological remodeling. Additionally, non-structural ECM components further amplify myofibroblast activation, exacerbating adverse remodeling (13).

Furthermore, even before the advent of single-cell sequencing technologies, the heterogeneity of fibroblasts and myofibroblasts had already been recognized (14). The sources of activated myofibroblasts extend beyond traditional fibroblasts, encompassing macrophages, endothelial cells, and bone marrow-derived cells. Both quiescent fibroblasts and activated myofibroblasts can be further subdivided into distinct subsets, expressing different gene markers and fulfilling diverse roles such as promoting inflammation, fibrosis, angiogenesis, or even anti-fibrotic functions (15). During this transformation, the formation of fibrotic scars, while initially protective, can lead to impaired cardiac function if the activation persists. A deeper understanding of these biological mechanisms could pave the way for novel therapeutic strategies targeting fibroblast activation and fibrosis to treat heart failure.

The integration of scRNA-seq with bulk RNA provides novel insights into disease progression by identifying novel cell subtypes, potential biomarkers and delineating intrinsic cell population heterogeneity (16). Furthermore, machine learning is an approach that leverages intricate algorithms to automatically analyze vast and diverse datasets, proving to be a valuable tool in bioinformatics big data analysis for assessing individual patient risks and treatment requirements (17). Moreover, Lasso regression is employed to address high-dimensional biomolecular features afflicted by multicollinearity (18). By utilizing integrated various algorithms, consensus models can be developed to predict prognosis, and offer more personalized evaluation approaches to inform clinical decision-making.

In this study, our objective is to establish a fibroblast landscape associated with IHD through integrated bioinformatics analysis, and identify novel disease targets and develop diagnostic models for heart failure patients with ischemic cardiomyopathy (ICM) and cardiac sarcoidosis. Additionally, we established a rat model of heart failure post-myocardial infarction to validate the observed gene expression changes. The findings of this research provide novel evidence for elucidating the distinct transcriptional profiles of IHD-related fibroblasts and uncovering the underlying biological mechanisms.

## Materials and methods

### Data source and acquisition

The dataset used in this study was obtained from the Gene Expression Omnibus (GEO) database (http://www.ncbi.nlm.nih.gov/geo), with the accession number GSE205734. This particular dataset comprises single nucleus RNA sequencing (snRNA-seq) data for IHD (3 samples) and cardiac sarcoidosis (CS, 4 samples) to identify distinct cell types and investigate their transcriptome profiles and regulatory mechanisms. The bulk RNA-seq datasets, with accession numbers GSE5406, and GSE57338, were also obtained from the GEO database. To characterize the gene expression profile of IHD, we recruited 108 IHD cases and 17 healthy donors from GSE5406, ensuring the availability of complete expression information. Differentially expressed genes (DEGs) were identified using the Limma package. For further validation, data from the GSE57338 dataset were extracted using identical inclusion criteria, comprising 95 patients diagnosed with IHD and 136 controls. All procedures pertaining to data acquisition and usage strictly adhered to the GEO database management policies.

### Clustering and dimensionality reduction classification of IHD-related fibroblast subtype

The raw data from GSE205734 comprises 28,360 cells from IHD samples and 29,147 cells obtained from cardiac sarcoidosis samples. We specifically analyzed the snRNA-seq data of cells expressing more than 200 genes but no more than 5,000 genes. To calculate the anchors, we utilized the Seurat package “FindIntegrationAnchors” function (reduction='cca') in R version 4.1.1.1. Subsequently, we integrated the gene expression data from multiple samples using the “IntegrateData” function. The resulting output was further employed for subsequent analysis and visualization.

The Uniform Manifold Approximation and Projection (UMAP) and the t-Distributed Stochastic Neighbor Embedding (t-SNE) methods were employed for dimension reduction. Afterwards, the cells were clustered into different subtypes using the “FindNeighbors” and “FindClusters” functions with the parameters dim ranging from 1 to 30 and resolution set at 0.5. The annotation of cell types was based on established marker genes reported in the early literature (19, 20). For further annotation of fibroblasts, we utilized six labeled genes (PDGFRA, PDGFRB, COL1A1, COL1A2, FAP, and DCN) and performed re-clustering and dimensionality reduction. To identify characteristic genes of each fibroblast subtype, we used the “FindAllMarkers” function with thresholds of logFC (multiple change) = 0.585, minpct = 0.25, and an adjusted *p*-value < 0.05. Additionally, we employed the clusterProfiler package to conduct gene and gene set enrichment analysis for ontologies and pathways.

### Signature derived from machine-learning-based ensemble methods

Acknowledging the critical role of activated fibroblasts in extracellular matrix formation and component modifications, we computed a Progressive Fibrosis Score (PFS) referring to Human Gene Set: THUM_MIR21_TARGET_HEART_DISEASE_UP obtained from MsigDB database for each fibroblast subtype utilizing the “AddModuleScore” function. We found that cluster2 exhibited the highest PFS compared with others.

Moreover, we identified the global characteristic genes of IHD using the bulk RNA sequencing data from the GSE5406 dataset. The Venn diagram method was employed to obtain the overlap between the DEGs of cluster2 and the global DEGs of IHD. This analysis aimed to assess the potential of characteristic genes within this subset of fibroblasts for the development of diagnostic tools for IHD. Then, the overlapping genes were tested for their association with IHD in a training cohort recruited from GSE5406 using a combination method of logistic regression and LASSO. Finally, an optimal gene panel consisting of 10 genes for constructing the IHD diagnosis tool was selected and validated in an independent IHD cohort obtained from the GSE57338 dataset. The nomogram was employed to visualize individualized evaluation models that were based on the 10 genes-derived diagnosis tool.

### H&E and masson staining

Cardiac tissue samples were collected from sham-operated and myocardial infarction (MI) rats 28 days post-operation. The tissues were fixed in 4% paraformaldehyde for 24 h at room temperature, dehydrated, embedded in paraffin, and sectioned into 5 μm thick slices. Hematoxylin-eosin (HE) staining (MXB Biotechnologies, China) was performed following standard protocols to evaluate general tissue structure. Masson's trichrome staining (Solarbio, China) was used to assess collagen deposition and fibrosis.

### Validated 10 genes by qPCR in heart failure ratmodels

For further validation, male Sprague-Dawley rats (8 weeks) were purchased from Guangxi Medical University Laboratory Animal Center. They were subjected to ligation of the left anterior descending coronary artery to survive for 28 days, establishing a rat model of heart failure post-myocardial infarction. The sham group underwent surgery without ligation. Total mRNA was extracted from the anterior wall of myocardial infarction tissues using TRNzol Universal Reagent (TIANGEN, Beijing, China) and then reverse-transcribed into cDNA (TIANGEN, Beijing, China), qPCR analyses were carried out with SYBR Green (TIANGEN, Beijing, China). The expression levels of mRNA were normalized to GAPDH using the 2^−*ΔΔ*Ct^ method.Primer sequences for the 10 featured genes are shown in Supplementary Table S2.

## Results

### Fibroblasts and endothelial cells are the predominant cellular components in the left ventricle of individuals with IHD

**Figure 1:**
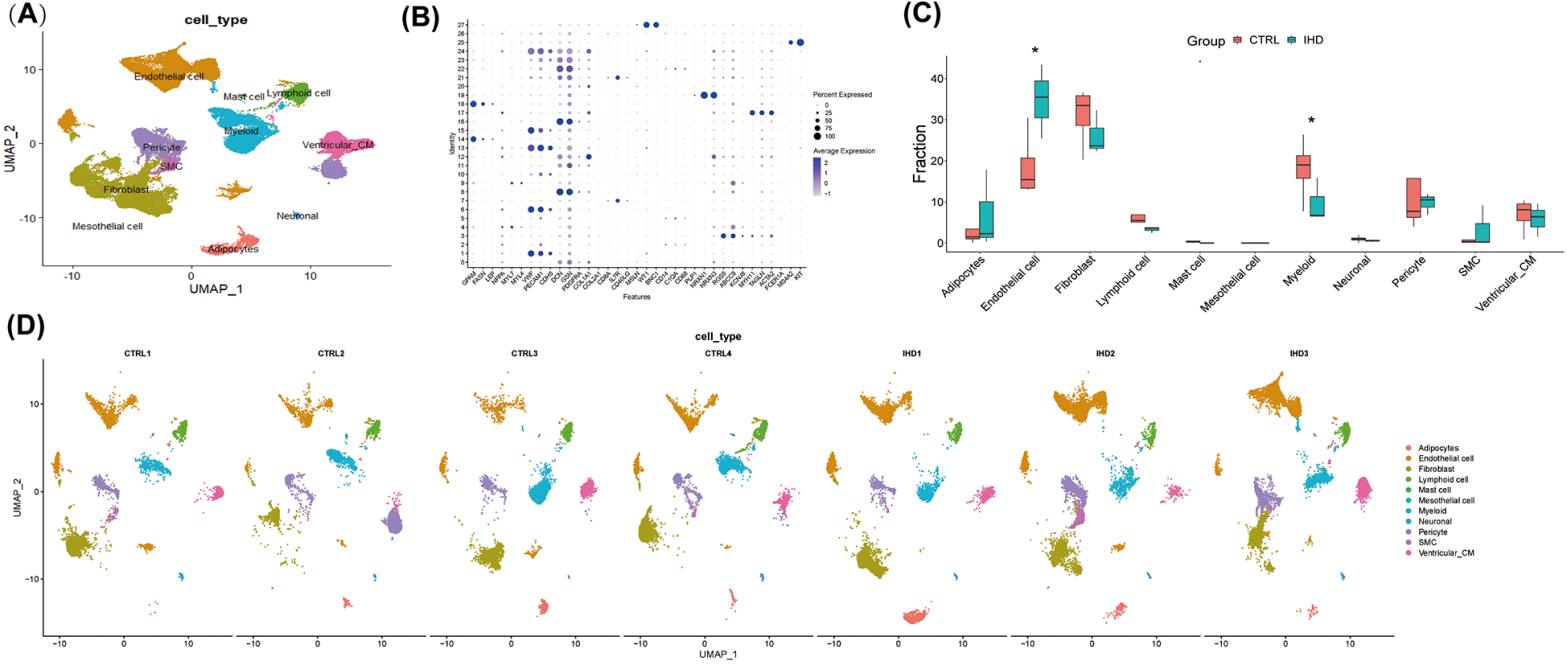
The cellular landscape of the left ventricular tissue in patients with ischemic heart disease (IHD) and cardiac sarcoidosis (CS). **(A)** UMAP visualization was used to display 11 annotated cell types in the hearts of individuals with IHD and control, utilizing color-coding and labels. **(B)** A dot plot was employed to illustrate the expression levels of marked genes used for annotating cell types, as well as the proportions of cells within each cluster expressing these genes. **(C)** The proportions of cell types in each group were color-coded according to their cell cluster. It was observed that the IHD group exhibited a higher proportion of endothelial cells and a lower proportion of myeloid cells compared to the CTRL group. **(D)** UMAP visualization was conducted to display the annotated cell clusters in each sample, demonstrating consistent projection positions of the same cell types.

In the experiment using this dataset (GSE205734), three samples of human left ventricle from individuals with ischemic heart disease (IHD) and four samples of cardiac sarcoidosis were subjected to single-nucleus RNA sequencing. The Seurat package in R was employed for data analysis, resulting in the identification of 53,419 high-quality cells based on the criteria of 500 < nFeatures < 4,000 and mitochondrial RNA percentage < 10%. The FindAllMarkers function was utilized to identify cluster-defining markers through differential expression analysis. To assign cell type identification to the clusters, an unbiased clustering approach was applied by comparing canonical markers previously reported in the literature (Figures 1A,B).

A total of 11 cell clusters were identified (Figure 1A), including cardiomyocytes, endothelial cells, fibroblasts, lymphocytes, myeloid cells, vascular smooth muscle cells, mast cells, stromal cells, pericytes, adipocytes, and neuronal cells. The results revealed that both groups primarily consisted of endothelial cells, fibroblasts, and myeloid cells (Figure 1C), which significantly differed from the cellular composition of non-diseased hearts, thereby highlighting the crucial role of these cells in elucidating the biological processes underlying the diseases. Notably, cells from other clusters exhibited well-defined clustering in the UMAP and showed no significant polarity, indicating the absence of a substantial batch effect between the two groups (Figure 1D).

### The *COL1A1*^hi^*MR4A1*^low^ fibroblast in IHD demonstrates a pronounced fibrotic phenotype

**Figure 2:**
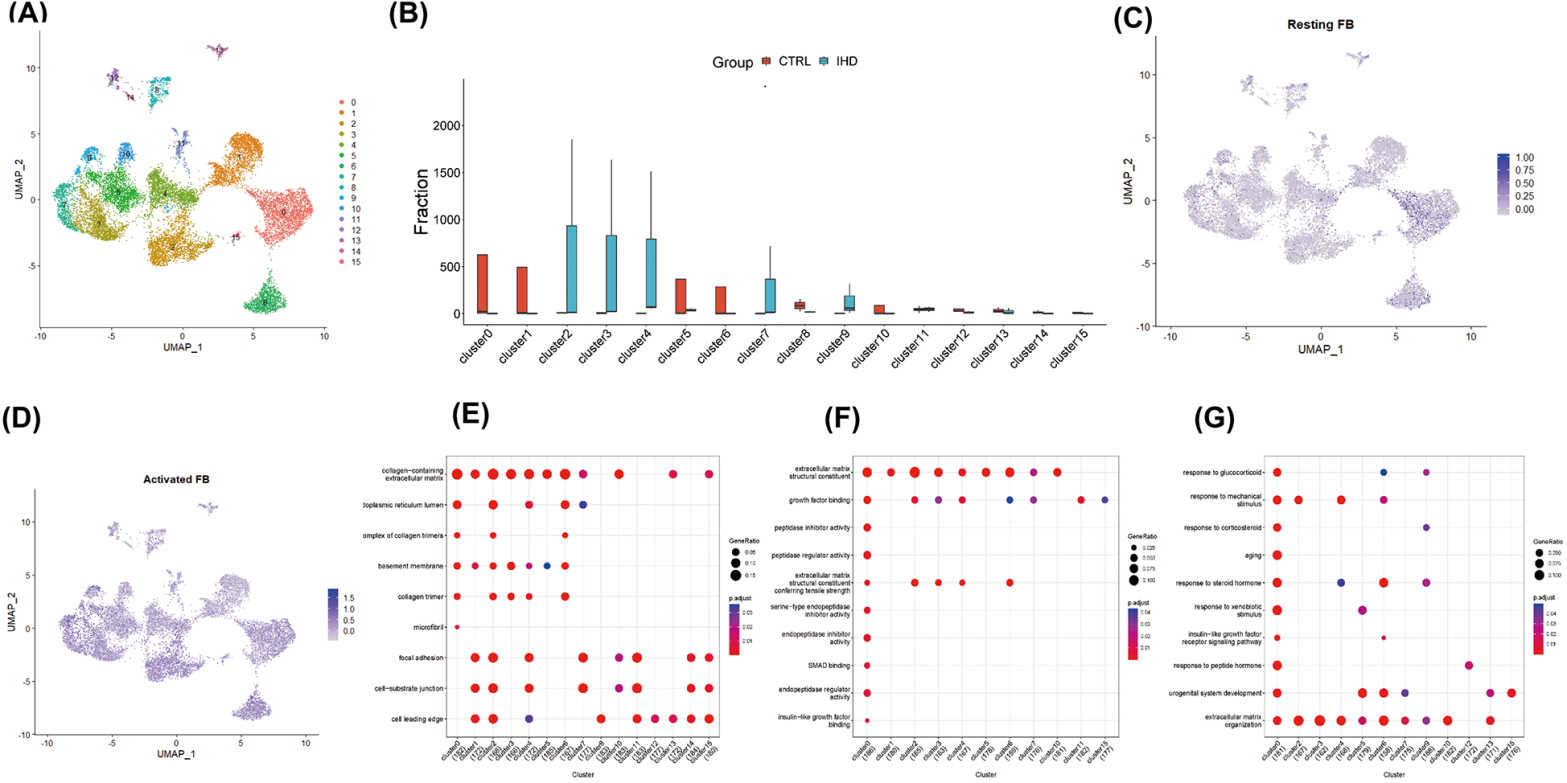
Identified and functionally characterized for fibroblast clusters. **(A)** The reclustering and visualization of 16 fibroblast lineages were performed using UMAP. **(B)** The comparison between different proportions of fibroblast subpopulations revealed significant heterogeneity in the composition of fibroblasts from different diseases. **(C)** UMAP demonstrated a reduced proportion of resting fibroblasts, identified by the expression of cell cycle inhibitory genes G0S2 and CDKN2C, as well as antioxidant genes GPX3 and GSTA3, within the global cell pool. **(D)** UMAP displayed a broad distribution of activated fibroblasts, marked by the expression of MMP3, RBP4, COL3A1, and C3, across various fibroblast clusters. Dot plots illustrated the enrichment analysis of characteristic genes in different fibroblast clusters, depicting the heterogeneity of biological pathways **(E)**, cellular components **(F)**, and molecular functions **(G).**

**Figure 3:**
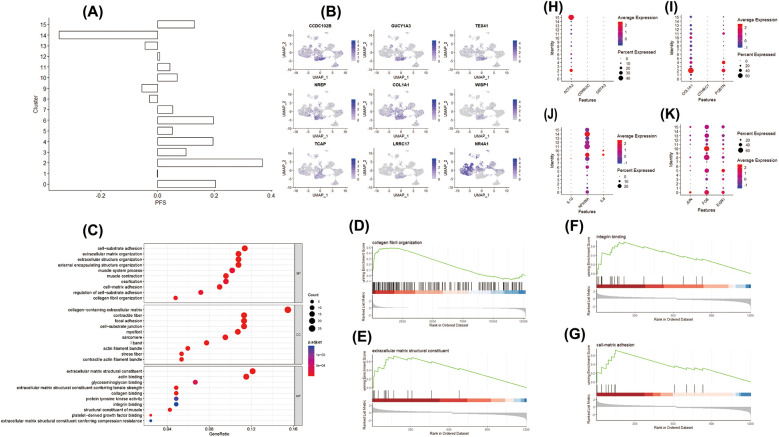
COL1A1^hi^NR4A1^low^ FB exhibited a strong correlation with progressive cardiac fibrosis. **(A)** The fibroblast clusters were assessed for their progressive fibrosis score using the human fibrosis gene dataset as a reference, revealing that cluster2 displayed the highest activity in the gene set. **(B)** UMAP visualization illustrated specific differentially expressed genes in cluster 2 fibroblasts. Notably, due to the elevated expression of WISP1, a key molecule in the classical fibrosis pathway, and the specific low expression of NR4A1, an endogenous inhibitor of the TGF-*β* pathway, this cluster was designated as COL1A1^hi^NR4A1^low^ FB. **(C)** Enrichment analysis demonstrated that the differentially expressed genes in COL1A1^hi^NR4A1^low^ FB were involved in various biological processes (top), cellular components (middle), and molecular functions (bottom) related to fibrosis. Gene set enrichment analysis (GSEA) further revealed that the expression patterns of gene sets associated with fibrosis, including collagen fibril organization **(D)**, extracellular matrix structural constituents **(E)**, integrin binding **(F)**, cell-matrix adhesion **(G)**, were closely related to the phenotype of COL1A1^hi^NR4A1^low^ FB. **(H)** markers of Rest fibroblast, **(I)** markers of activated Myofibroblast, **(J)** markers of pro-imflammation fibroblast, **(K)** markers of transitional Myofibroblast.

Given the central role of fibroblast expansion and activation in the progression of cardiac fibrosis, fibroblast clusters were extracted for further analysis. By applying the aforementioned method, fibroblasts were reclustered, resulting in the generation of 16 cell clusters at a resolution of 0.8, based on distinctive gene expression profiles (Figures 2A,B). To characterize their functional state, we employed the labels of cell cycle inhibitory genes G0S2 and CDKN2C, along with antioxidant genes GPX3 and GSTA3, to identify resting fibroblasts. The findings revealed a minimal proportion of resting fibroblasts in both IHD and CS hearts, while activated fibroblasts exhibiting high expression of MMP3, RBP4, COL3A1, and C3 were predominantly observed (Figures 2C,D). To further delineate fibroblast functions, we used previously identified subgroup markers (15): high CDKN2C and low COL1A1/COL3A1 for resting fibroblasts, CHTRC1, COL1A1, and COL3A1 for myofibroblasts, NFKBIA, IL10, and IL6 for inflammatory fibroblasts, and FOS, JUN, and EGR1 for transitional states. The analysis indicated that clusters 6, 8, 10, and 13 corresponded to resting fibroblasts, clusters 2 and 4 to activated myofibroblasts, clusters 9 and 14 to inflammation-associated fibroblasts, and clusters 0, 5, 10, and 15 as transitional myofibroblasts (Figures 3H–K). Further analysis demonstrated that clusters 2, 3, 4, 7, and 9, predominantly originating from IHD samples, were enriched in biological processes such as extracellular matrix organization, external encapsulation structure organization, cell matrix adhesion transmembrane receptor, as well as functions involved in actin binding, collagen binding, DNA binding, and transcription factor binding (Figures 2E–G).

These findings implied a strong link between fibroblasts, extracellular matrix remodeling, and fibrosis in the context of IHD. Thus, we utilized the 18 genes associated with cardiac fibrosis from the MsigDB database Human Gene Set: THUM_MIR21_TARGET_HEART_DISEASE_UP to evaluate the pro-fibrotic potential of different fibroblast clusters in the upstream analysis. The average expression levels of these genes were analyzed using the AddModuleScore function from the Seurat package, enabling the calculation of the progressive fibrosis score (PFS) for each cluster. As depicted in Figure 3B, the fibroblast subpopulation in Cluster2, primarily originating from IHD, exhibited the highest progressive fibrosis score (PFS), indicating heightened activity of its fibrosis-related gene set. By employing the parameters of an adjusted *p*-value ≤ 0.05 and |log2 Foldchange| ≥ 1.0, we identified 108 DEGs in Cluster2. Importantly, Cluster2 exhibited elevated expression of COL1A1, GUCY1A3, CCDC102B, TEX41, NREP, WISP1, TCAP, LRRC17 and minimal expression level of NR4A1 (Figure 3B). COL1A1 is one of the most important extracellular matrix components in fibrotic heart. NR4A1, an important endogenous TGF-*β* pathway inhibitor, were observed to be downregulated in this cell cluster specifically. To facilitate the understanding of the molecular characteristics of Cluster2, we designated this cluster as COL1A1^hi^NR4A1^low^ fibroblasts (COL1A1^hi^NR4A1^low^ FB). Additionally, COL1A1^hi^NR4A1^low^ FB featured genes were enriched in biological processes related to cardiac fibrosis and its regulation pathway (Figure 3C). The GSEA analysis revealed a gene set enrichment of COL1A1^hi^NR4A1^low^ FB in pathways associated with collagen fibril organization, extracellular matrix structural constituent, integrin binding, cell-matrix adhesion, cytoskeletal protein binding, and cell substrate adhesion (Figures 3D–I). To further explore the potential regulators of NR4A1 in the context of IHD, we searched the UCSC Genome Browser website and found that the TCF4, a negative regulatory factor of NR4A1 transcription, is upregulated in COL1A1^hi^NR4A1^low^ FB. Furthermore, prediction analysis using the JASPAR database revealed that TCF4 is capable of binding to the NR4A1 promoter at 16 different sites, with a relative profile score threshold of 80%. Notably, the site starting at 1,140 base pairs and ending at 1,147 base pairs exhibits the highest binding score of 9.758579 for the TCF4 and NR4A1 promoter interaction (Supplementary Table S1). Additionally, COL1A1^hi^NR4A1^low^ fibroblasts exhibit elevated expression of other myofibroblast markers such as POSTN and ACTA2 (Supplementary Figures S1, S2, and Table S3), These findings highlight the potential of this fibroblast subset to actively contribute to the progression of cardiac fibrosis in patients with IHD.

### Diagnostic performance testing and *in vivo* validation of *COL1A1*^hi^*NR4A1*^low^ fibroblast characteristic genes for IHD

**Figure 4:**
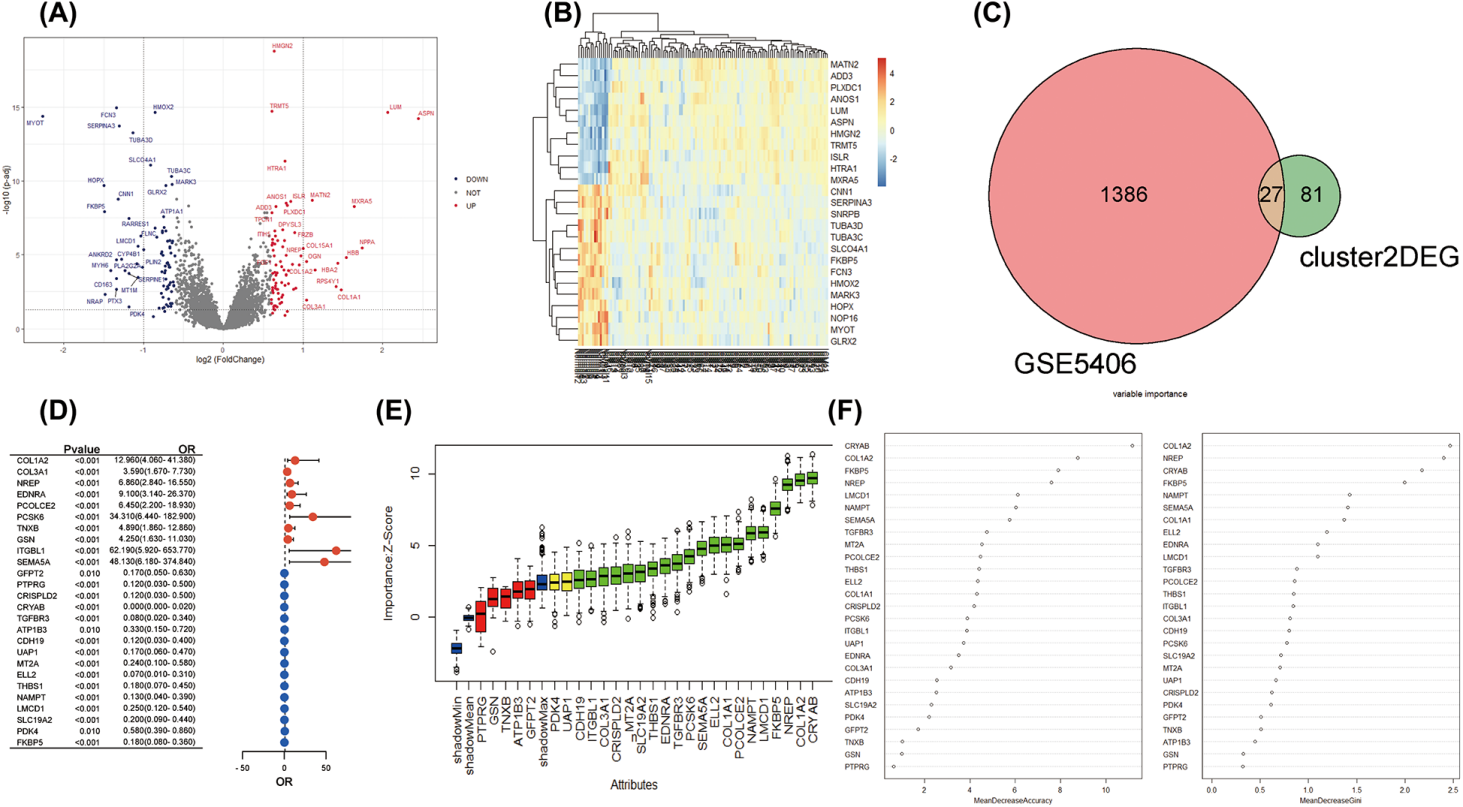
The integration analysis of single-cell and bulk RNA sequencing revealed the strong association between COL1A1^hi^NR4A1^low^ FB characteristic genes and IHD. **(A)** In GSE5406, 108 IHD and 17 non-heart failure subjects were recruited. Volcano plots were used to display the global differences in cardiac RNA expression, resulting in 714 upregulated genes and 699 downregulated genes in IHD hearts. **(B)** Unsupervised clustering analysis based on gene expression levels revealed intra-group similarity and reliable inter-group differences between the two groups of heart samples. **(C)** The Venn method was used to identify 27 overlapping genes between the characteristic expressed genes of Fibroblast cluster2 (COL1A1^hi^NR4A1^low^ FB) derived from IHD mainly and the globally differentially expressed genes in IHD. **(D)** A forest plot was displayed, showing logistic regression (adjusted for age and sex) exploring the association between the 27 genes and IHD. **(E)** Machine learning based on the Boruta algorithm was used to evaluate the importance of individual genes. Twenty important genes (Confirmed variables, with importance Z-scores higher than those of shadowMax) were filled in green, two potentially important genes (Tentative variables, with importance Z-scores equal to those of shadowMax) were filled in yellow, and five unimportant genes (Rejected variables, with importance Z-scores lower than those of shadowMax) were filled in red. **(F)** A machine learning model based on the random forest algorithm was used to calculate the importance of the 27 genes and sort them in descending order.

Moreover, we explored the bulk gene expression profile of IHD based on RNA sequencing data from left ventricular myocardium samples obtained from 108 IHD cases and 17 healthy donors. A total of 1,413 DEGs were identified for further analysis. Among them, 714 genes were found to be up-regulated in IHD, while 699 genes showed down-regulation (Figure 4A). Unsupervised clustering analysis effectively distinguished IHD cases from healthy hearts based on these DEGs (Figure 4B). Furthermore, we utilized the Venn method to identify 27 common differentially expressed genes when mapping with characteristic genes of *COL1A1^hi^NR4A1*^low^ FB (Figure 4C). Given the potential significance of *COL1A1^hi^NR4A1*^low^ FB in IHD cardiac fibrosis, we further investigated the association with the disease and importance of these 27 genes using univariate logistic regression, the Boruta algorithm, and the random decision forest model. Among the various models, CRYAB, COL1A2, NREP, FKBP5, and LMCD1 exhibited the strongest association and importance for IHD (Figures 4D–F).

**Figure 5:**
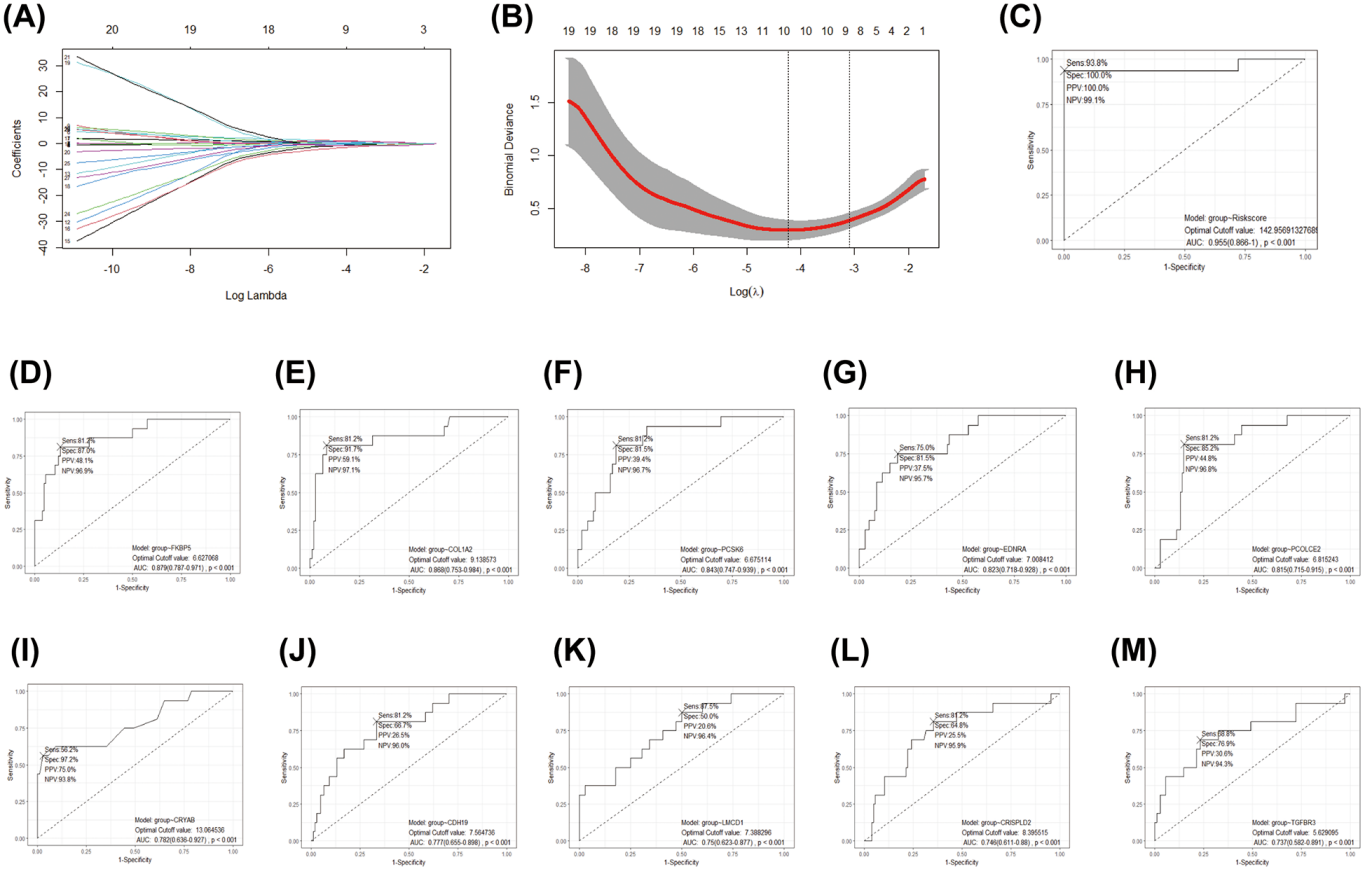
Development and efficacy evaluation of an IHD diagnostic model based on COL1A1^hi^NR4A1^low^ FB feature genes. **(A)** The coefficient path diagram of the LASSO regression model depicted the trajectory of the coefficient change for each independent variable (one of the 27 genes). The *y*-axis represented the coefficient value, and the *x*-axis represented the log(*λ*) value. **(B)** The cross-validation diagram of the LASSO regression model displayed the parameter of log(*λ*) and Deviance. The dashed line on the left corresponded to the smallest Deviance (optimal model), resulting in the retention of 10 genes. The dashed line on the right corresponds to the simplest model within one standard deviation, resulting in the retention of 8 genes. **(C)** The effectiveness of the optimal gene features selected by LASSO regression as diagnostic methods for IHD was evaluated by calculating the risk score using The “points cal()” function from the nomogramFormula package in R. **(D–M)** The diagnostic efficacy of individual genes within the optimal gene features screened by the LASSO model for IHD.

A LASSO logistic regression model was employed to address the potential multicollinearity among these 27 genes. As a result, the best diagnostic features for IHD were identified among 10 genes, including COL1A2, EDNRA, PCOLCE2, PCSK6, CRISPLD2, CRYAB, TGFBR3, CDH19, LMCD1, and FKBP5 (Figures 5A–B). We developed a diagnostic method for IHD based on a logistic regression model utilizing 10 selected *COL1A1*^hi^*MR4A1*^low^ FB marked genes. The “points cal()” function from the nomogramFormula package was used to calculate the score for each individual based on the 10 genes mentioned above, which was defined as the RiskScore. Subsequently, the RiskScore was tested as a new variable for diagnosing IHD. The receiver operating characteristic (ROC) curve demonstrated that the area under the curve (AUC) for the new variable was 0.955 (95% CI 0.866–1.000), with an optimal cutoff value of 142.96 (Figure 5C). These findings suggest that the RiskScore calculated using the 10 *COL1A1*^hi^*MR4A1*^low^ FB gene features may exhibit good discriminative ability for IHD. Additionally, FKBP5 displayed the highest discriminative ability (AUC = 0.879) among the individual molecules for IHD, followed by COL1A2 (AUC = 0.868), PCSK6 (AUC = 0.843), EDNRA (AUC = 0.823), and PCOLCE2 (AUC = 0.815).

**Figure 6:**
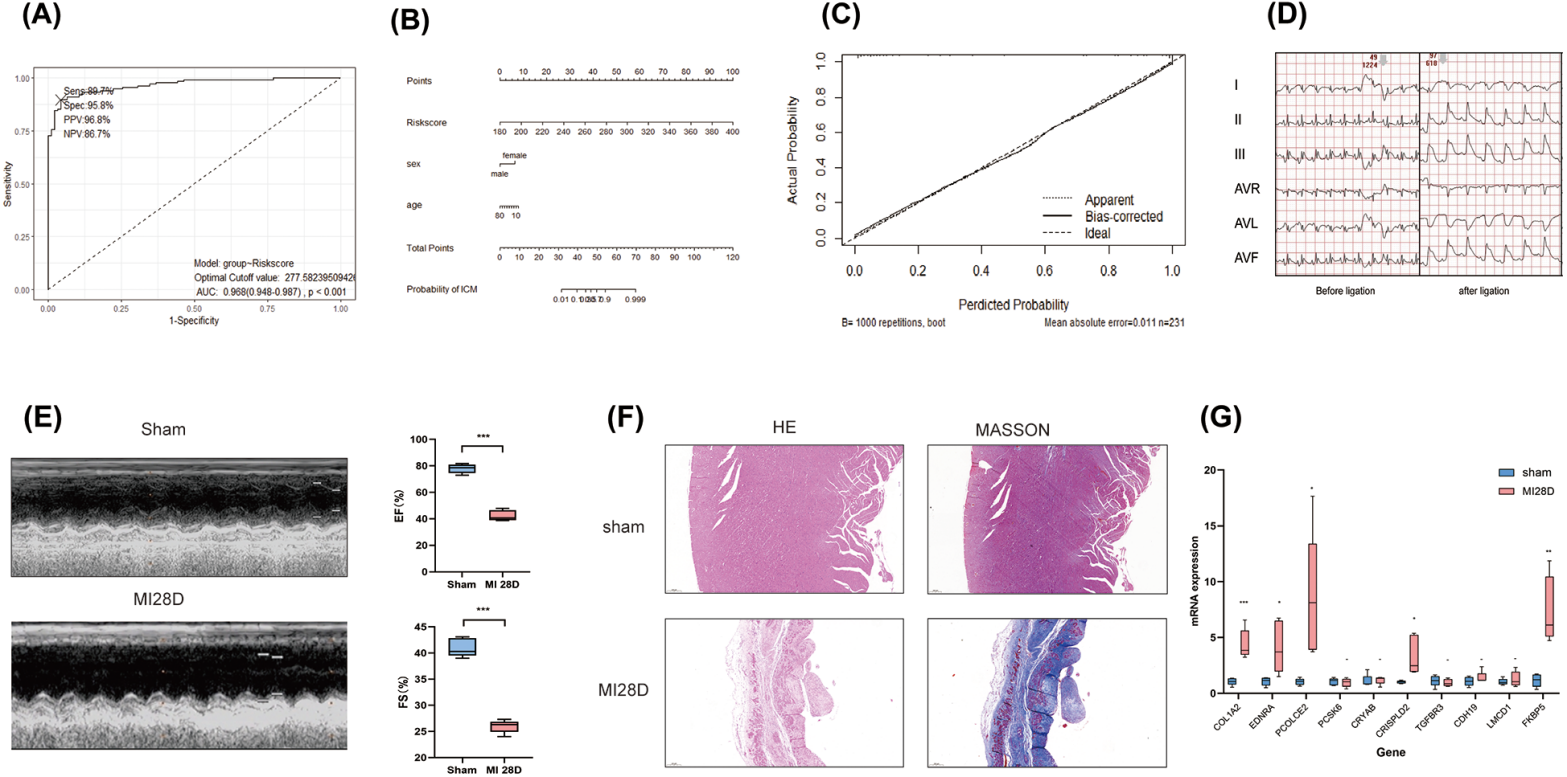
External validation of diagnostic models based on COL1A1^hi^NR4A1^low^ FB feature genes was conducted. **(A)** 95 cases of IHD and 136 control populations recruited from the GSE57338 dataset were used as external cohort to validate the robustness of the 10-genes-derived RiskScore previously developed for IHD diagnosis. **(B)** A nomogram was presented to display the contribution of RiskScore in the diagnosis of IHD. **(C)** The calibration curve illustrated a favorable correspondence between the model predictions based on RiskScore and the observed probabilities. **(D)** Electrocardiographic in rats pre-ligation, immediately post-ligation**(E)** Echocardiographic analysis comparing cardiac function between sham-operated rats and those subjected to LAD ligation 28 days post-surgery. **(F)**H&E and Masson staining of myocardial tissue from sham-operated and post-MI rats at 28 days post-operation rat. **(G)** Quantitative PCR validation of mRNA levels for 10 characteristic genes in infarcted myocardial tissue from rats 28 days post-myocardial infarction (MI) compared to sham controls. *n* = 5 (Independent sample *t*-test was used to compare the differences between the two groups, *P < 0.05, **P < 0.01, ***P < 0.001,).

To validate the performance of the model, an independent validation was conducted. We recruited a total of 95 patients diagnosed with IHD and 136 controls without heart failure from the GSE57338 dataset. Similar to the training cohort, the left ventricular tissue samples from these individuals were subjected to bulk RNA sequencing using the Affimetrix Human Gene Array platform. This approach was implemented to control for possible measurement bias. Utilizing the same methodology for calculating the *COL1A1^hi^NR4A1*^low^ FB gene features score for each participant, the ROC curve consistently demonstrates the high discriminatory capacity of RiskScore for identifying IHD in this independent cohort, with an AUC of 0.968 (Figure 6A). Furthermore, the nomogram indicated that RiskScore exhibited superior discriminatory ability compared to age and sex (Figure 6B). The calibration curve illustrated an agreeable correspondence between the model predictions based on RiskScore and the observed probabilities (Figure 6C), which were validated through 1,000 replicates to account for bias.

Finally, we validated the expression of 10 previously mentioned genes in a rat model of heart failure, 28 days post-myocardial infarction (MI). Electrocardiographic changes confirmed the successful induction of the acute myocardial infarction (AMI) model (Figure 6D). Similarly, echocardiographic assessments demonstrated the effective establishment of the ischemic heart failure model in rats (Figure 6E). qRT-PCR results showed that, compared to the sham group, the mRNA expression levels of the 10 genes in the myocardial infarction tissue of rats 28 days post-MI were elevated; five of these genes, including COL1A2, EDNRA, PCOLCE2, CRISPLD2, and FKBP5, exhibited significant increases (Figure 6F). These findings further support the results from the bulk RNA sequencing analysis.

## Discussion

The progression of cardiac fibrosis is the primary factor contributing to the decline in cardiac function and the development of heart failure in individuals with IHD (21). While, reversing fibrosis as a direct treatment for heart failure requires identifying specific biomarkers and feasible therapeutic targets. This study utilized an integrated approach that combined single-cell and bulk transcriptomic analysis, as well as machine learning algorithms, to reveal the heterogeneity of fibroblasts in various cardiac fibrotic diseases and screen potential biomarkers for IHD within highly activated fibroblast subset. Our findings demonstrated the number of fibroblasts in IHD heart samples significantly increased and was less in a resting state, indicating that the expansion of the fibroblast pool and functional activation may be key regulatory factors for IHD induced cardiac fibrosis. Furthermore, we found a robust correlation between the COL1A1^hi^NR4A1^low^ FB cluster obtained from the hearts of individuals with IHD and the progression of cardiac fibrosis. Moreover, the characteristic expression of a COL1A1^hi^NR4A1^low^ FB -derived 10-gene panel could be utilized as a reliable diagnostic method for IHD.

Previous studies have reported that the cardiac extracellular matrix (ECM) primarily consists of type I and III collagen, with lower abundance of types IV, V, and VI collagen (22). Type I collagen constitutes approximately 85% of the total myocardial collagen and is responsible for constructing coarse fibers that provide tensile strength for heart. Type III collagen, which accounts for 11% of the total collagen in the normal heart, is assembled as delicate fibers to regulate the elasticity of the matrix network (23). In the process of cardiac fibrosis, the accumulation of type I collagen derived by activated fibroblasts and is the main contributor to increased cardiac stiffness and impaired function (24, 25). The significance of fibroblasts in the development of cardiac fibrosis is well-documented due to the involvement of extensive signaling pathways and their intricate interactions (26, 27). Fibrogenic growth factors, such as TGF-*β*_1_ and PDGF, as well as neurohormonal factors like angiotensin II and aldosterone, along with endothelin-1, play a pivotal role in the pathogenesis of myocardial fibrosis (21). Notably, the activation of the TGF-*β* pathway in cardiac fibroblasts is of particular importance as it induces and sustains an activated fibroblast phenotype, ultimately leading to the transcription and translation of COL1A1, COL1A2, and other genes related to fibrosis (28). This activation occurs via both the classical Smad2/3 dependent pathway and the alternative Rho/Rho-related protein kinases (ROCK) pathway (29, 30). However, the functional status of fibroblasts is regulated by a complex interplay of positive co-stimulatory and negative co-inhibitory signals. During normal wound healing, there is an instantaneous increase in TGF-*β* signal transduction, which activates fibroblasts. Once the repair process is complete, TGF-*β* signal transduction is terminated and extracellular matrix synthesis returns to normal levels (31). However, in fibrotic diseases, the weakening of TGF-*β* signal transduction does not occur, resulting in sustained signal transduction that leads to chronic activation of fibroblasts and significant accumulation of extracellular matrix (32). The molecular mechanism underlying the failure to restrict TGF-*β* activity remains unclear.

Recently, it has been observed that co-inhibitory signals of the TGF-*β* pathway, such as NR4A1 (33), Del 1 (34), RAC1 (35) are dysfunctional or depleted in response to tissue damage or chronic stimulation. Nevertheless, the underlying regulatory mechanisms governing this process remain poorly understood. In the present study, we re-clustered fibroblasts from heart samples of individuals with IHD and CS, resulting in the identification of 16 clusters with distinct transcriptional profiles. COL1A1^hi^NR4A1^low^ fibroblasts subset shows high expression of typical activated myofibroblast markers like COL1A1 and CHTRC1, suggesting its significant potential in promoting cardiac fibrosis. Myofibroblasts are a conditionally induced heterogeneous cell population (36), and COL1A1^hi^NR4A1^low^ FB maybe represent a critical subpopulation playing a key role within the myofibroblast group. We further utilized a reference set of human progressive fibrosis-related genes to quantitatively assess and compare the likelihood of cardiac fibrosis among different fibroblast subsets. Remarkably, the COL1A1^hi^NR4A1^low^ FB subset demonstrated the highest level of activity in the fibrotic gene set, suggesting a strong association with cardiac fibrosis.

In the present study, we focused on a downregulation of the endogenous TGF-*β* signaling pathway inhibitor, NR4A1, within this subgroup of fibroblasts. It has been reported that NR4A1 can form a complex with SP1 and bind to the COL1A1 promoter (−242 bp, binding site 6/7), thereby inhibiting its transcription induced by Smads (33). Furthermore, the TCF4, a negative regulatory factor of NR4A1 transcription, is upregulated in COL1A1^hi^NR4A1^low^ FB. TCF4 has been reported to undergo upregulation in response to hypoxic conditions and participate in the activation of either the Wnt pathway or the HIF-1*α* signaling pathway, both of which play crucial roles in cellular proliferation and differentiation (37, 38). These findings suggest that the increased activation of COL1A1^hi^NR4A1^low^ FB may be attributed, at least in part, to the diminished TGF-*β* signaling induced by downregulated NR4A1.

We also found this specific subset of fibroblast exhibited elevated expression of known profibrotic factors, including mechanoreceptor PIEZO2 (39) and related pathway regulator GUCY1A3 (40),CRYAB (41), PCSK6 (42),THBS2 (43) and VCAN (44)that initiating intracellular synthesis and secretion of matrix proteins respond to mechanical stress and ang II stimulation,This is consistent with previous findings (45), which also reported the upregulation of THBS2 and VCAN in the context of fibrosis. More importantly, the potential but unconfirmed ability of TGFBR3, LMCD (46), and FKBP5 (47, 48) to mediate TGF-*β* pathway signaling in ischemic or hypoxic environments warrants further exploration in preclinical and clinical research. Moreover, the decreased expression of TGFBR3 in this subcluster compromised its capacity to inhibit the phosphorylation of Erk1/2, JNK, Smad2, and Smad3, as well as the transcription of genes associated with fibrosis (49–51). Our findings highlight the critical role of TGF-*β* signaling in fibroblast activation and cardiac fibrosis progression, providing new molecular insights for a deeper understanding of its upstream regulatory mechanisms.

To further investigate the IHD diagnostic significance of the COL1A1^hi^NR4A1^low^ FB, we conducted an evaluation of the diagnostic efficacy of characteristic genes associated with this fibroblast subtype by integrating bulk RNA data from two independent cohorts. By employing different machine learning models, we performed a comprehensive analysis and identified 10 COL1A1^hi^NR4A1^low^ FB signature genes (COL1A2, EDNRA, PCOLCE2, PCSK6, CRISPLD2, CRYAB, TGFBR3, CDH19, LMCD1, and FKBP5), which have a strong discriminatory potential for IHD and healthy populations. Subsequently, we developed a gene scoring tool (referred to as RiskScore) that utilizes the expression levels of 10 genes to facilitate the diagnosis of IHD. Notably, in an independent cohort, this tool demonstrated robust diagnostic efficacy and exhibited a more pronounced contribution compared to age and sex.In line with prior findings in the present study (52), these genes have been implicated in pathways involved in immune cell-fibroblast interaction, epithelial-mesenchymal transition, fibroblast proliferation, and activation of classical fibrotic gene transcriptional programs. Finally, considering that the post-myocardial infarction heart failure is a well-established *in vivo* model for translating IHD, we validated the expression of 10 signature genes in rat post-myocardial infarction heart failure model. The results indicated that the expression changes of 5 of these genes were consistent with our bulk RNA sequencing data.The identification of COL1A1^hi^NR4A1^low^ FB and their associated pro-fibrotic gene signatures provides novel insights into the mechanisms of cardiac fibrosis in IHD, not only delineates the transcriptomic heterogeneity of fibroblasts but also facilitates the development of a diagnostic model that could potentially improve the early identification and treatment of IHD-related fibrosis.

The identified biomarkers, particularly those linked to the COL1A1^hi^NR4A1^low^ fibroblast subset, might be helpful for further research of etiology, diagnosis and treatments. Invasive methods, such as endomyocardial biopsy, could leverage these markers to assess myocardial fibrosis severity, enabling precise diagnosis and guiding antifibrotic therapies. Additionally, their potential detectability in peripheral blood offers a less invasive approach for early detection, disease monitoring, and therapy evaluation.

Transcriptomic analyses showed high specificity and sensitivity for these biomarkers, supported by robust AUC values. Preliminary validation in a myocardial infarction-induced heart failure rat model further confirmed significant differential expression of several genes, highlighting their relevance in fibrosis. However, further studies using molecular assays, such as ELISA or qPCR, are needed to confirm their detectability and stability in blood samples. Standardization of thresholds and large-scale validation are crucial to ensure broader clinical applicability.

Our study contains limitations that merit attention. Firstly, the case-control design used for the bioinformatics analysis limits the ability to establish causal relationships, highlighting the need for future prospective studies and multidimensional experiments. Secondly, our study focused solely on investigating the biological mechanisms underlying reactive cardiac fibrosis in ischemic heart disease. Additionally, the low proportion of cardiomyocytes detected in the scRNA-seq data likely attributable to the severe cardiac dysfunction in the original samples (ejection fractions of 10%, 15%, and 20%) from end-stage heart failure patients, with fibrotic tissue predominating, Additional human cardiac samples should be analyzed and validated in future studies. Consequently, these findings may not be directly applicable to early replacement fibrosis, a crucial aspect in preventing cardiac rupture and other mechanical complications.

## Conclusion

In conclusion, this study identified a fibroblast subcluster, COL1A1^hi^NR4A1^low^ FB, strongly associated with cardiac fibrosis in IHD. A robust assessment tool based on the characteristic genes of this specific fibroblast cluster was developed for IHD diagnosis. Our findings could contribute to a better understanding of the role of the TGF-*β* signaling pathway in fibroblast activation and emphasize the promise of exploring novel ways to regulate this pathway in the development of new strategies for preventing and treating heart failure.

## Data Availability

The datasets presented in this study can be found in online repositories. The names of the repository/repositories and accession number(s) can be found in the article/Supplementary Material.
