# Supplementary figures and images for: Enhanced fibrotic potential of COL1A1hiNR4A1low fibroblasts in ischemic heart revealed by transcriptional dynamics heterogeneity analysis at both bulk and single-cell levels
Source: Front Cardiovasc Med. 2025 Jan 6;11:1460813. doi: 10.3389/fcvm.2024.1460813 (PMC11743554; doi:10.3389/fcvm.2024.1460813)

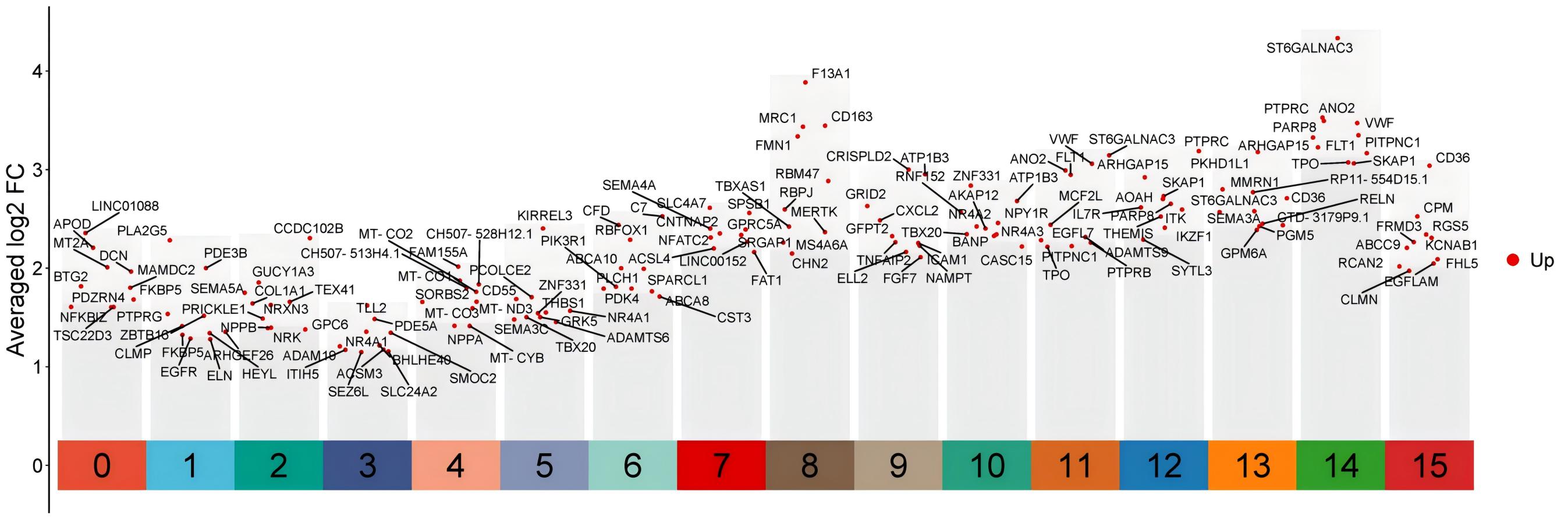

Supplement: Supplementary file 2 [file Image1.jpeg]

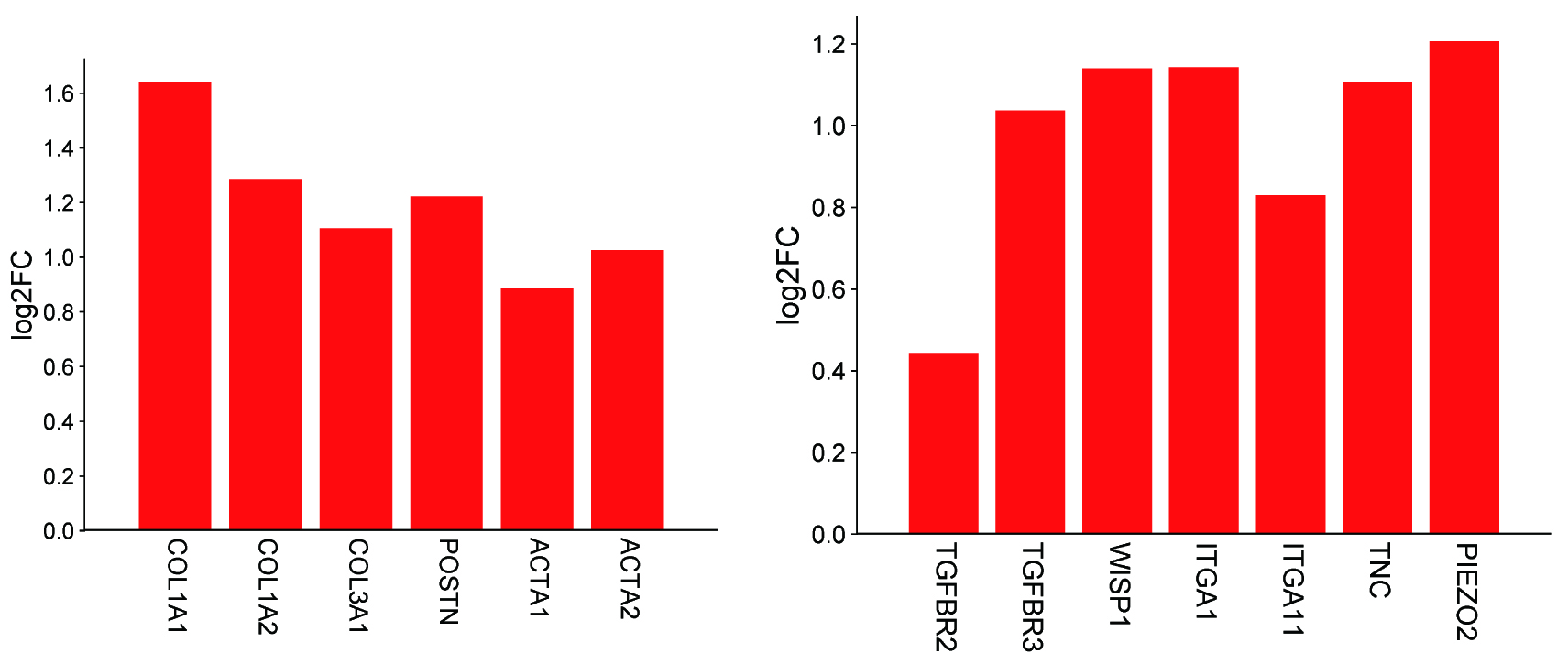

Supplement: Supplementary file 3 [file Image2.jpeg]
